# 晚期非小细胞肺癌*MET*扩增介导获得性耐药的研究进展

**DOI:** 10.3779/j.issn.1009-3419.2022.102.23

**Published:** 2022-08-20

**Authors:** 思思 潘, 娜 王, 霞 宋

**Affiliations:** 1 030001 太原，山西医科大学第二临床医学院 The Second Clinical Medical College of Shanxi Medical University, Taiyuan 030001, China; 2 030013 太原，山西省肿瘤医院呼吸二病区 The Second Department of Respiratory, Shanxi Provincial Cancer Hospital, Taiyuan 030013, China

**Keywords:** 肺肿瘤, 靶向治疗, *MET*扩增, 获得性耐药, Lung neoplasms, Targeted therapy, *MET* amplification, Acquired resistance

## Abstract

间质-上皮细胞转化因子（mesenchymal-epithelial transition factor, *MET*）扩增是表皮生长因子受体（epidermal growth factor receptor, EGFR）阳性非小细胞肺癌（non-small cell lung cancer, NSCLC）耐药的重要驱动因素，MET-酪氨酸激酶抑制剂（tyrosine kinase inhibitors, TKIs）联合EGFR-TKIs的治疗策略可以克服MET介导的获得性耐药。研究表明，*MET*扩增也是间变性淋巴瘤激酶（anaplastic lymphoma kinase, *ALK*）、*RET*、*ROS1*等驱动基因阳性NSCLC患者接受TKIs类药物治疗后耐药的驱动因素。本文综述了近年来关于*MET*扩增作为驱动基因阳性NSCLC靶向治疗耐药驱动因素的研究进展，并总结了克服这种耐药机制的治疗策略。

## Abstract (en)

Mesenchymal-epithelial transition factor (*MET*) amplification is an important driver of resistance in epidermal growth factor receptor (*EGFR*)-mutant non-small cell lung cancer (NSCLC), and the combination of MET proto-oncogene (*MET*) and EGFR-tyrosine kinase inhibitors (TKIs) has shown promise in overcoming this molecularly defined acquired resistance. Emerging data also demonstrate *MET* amplification as a resistance driver to TKIs-treated anaplastic lymphoma kinase (*ALK*)-, *RET*-, and *ROS1*-fusion NSCLC. Here, we review the literature on recent research progress of *MET* amplification as a resistance driver to targeted therapy in oncogene-driven NSCLC and summarize the progress of clinical strategies to overcome the resistance mechanism.

如今，针对驱动基因变异的靶向治疗药物已经为恶性肿瘤患者带来了长期获益和生存，近年来靶向治疗因其独特的作用机制、突破性的疗效和较少的不良反应也一直是肿瘤药物研发的方向，然而，随之而来的便是靶向治疗耐药的问题，解决酪氨酸激酶抑制剂（tyrosine kinase inhibitors, TKIs）类药物的耐药问题是精准医学时代的关键挑战。一些证据表明，间质-上皮细胞转化因子（mesenchymal-epithelial transition factor, *MET*）扩增是表皮生长因子受体（epidermal growth factor receptor, *EGFR*）等驱动基因阳性非小细胞肺癌（non-small cell lung cancer, NSCLC）患者接受TKIs类药物治疗后获得性耐药的一致性分子机制，双靶联合治疗策略有望可以克服*MET*扩增介导的获得性耐药。本文就*MET*基因及其信号通路、*MET*扩增的检测、获得性耐药现状及其联合治疗策略进行了综述。

## 1 *MET*基因和肝细胞生长因子（hepatocyte growth factor, HGF）/MET信号通路

*MET*基因，位于人类7号染色体长臂（7q21-31）上，全长约125 kb，由21个外显子和20个内含子组成，其编码分子量约150 kDa的c-MET蛋白前体，经局部糖基化后生成一个190 kDa的糖蛋白，该糖蛋白进一步剪切为α亚基（50 kDa）和β亚基（140 kDa），两者以二硫键连接形成一个异源二聚体-酪氨酸激酶受体，即为c-MET蛋白，也被称为肝细胞生长因子受体（hepatocyte growth factor receptor, HGFR）^[1]^。

HGF是目前发现的c-MET的唯一配体，是间质细胞在发育过程中产生和分泌的旁分泌信号分子。成熟的活性HGF是由α链和β链通过二硫键形成的二聚体。只有当c-Met和HGF的α链完全结合时，才能完全诱导HGF/MET信号通路的激活^[2]^。在生理状态下，HGF/MET信号通路的激活，负责调控多种生物学过程，包括细胞发育、增殖等，这对于多细胞生物体的完整性至关重要^[2]^。在胚胎发育过程中，MET-HGF结合可以促进肌肉生长和神经系统的形成^[3]^。在成年生物体内，它具有促进伤口愈合、组织修复及阻碍器官纤维化的生物学功能^[3]^。而HGF/MET信号通路的失调不仅可以诱导肿瘤的发生，而且还会导致肿瘤的侵袭、增殖、血管生成和转移扩散等^[4]^。c-MET通路的异常激活主要包括三种类型：*MET*外显子14跳跃突变、*MET*扩增和MET蛋白过表达^[5]^。

## 2 *MET*扩增

### 2.1 定义

*MET*扩增可以通过多倍体和局部扩增两种方式来实现。携带*MET*基因的7号染色体出现全基因组异常复制时就会形成多倍体，多倍体除了会导致*MET*基因拷贝数（gene copy number, GCN）增加，也会使得位于7号染色体上其他基因（如*EGFR*等）的拷贝数平行增加，而局部扩增则不会因为整条染色体的复制使得其他基因的拷贝数增加^[6]^。

### 2.2 检测方法

目前，对于*MET*扩增的检测方法有很多，包括荧光原位杂交（fluorescence *in situ* hybridization, FISH）、二代测序（next-generation sequencing, NGS）、实时定量聚合酶链反应（quantitative real time polymerase chain reaction, qRT-PCR）和免疫组织化学（immunohistochemistry, IHC）等^[7]^。然而，上述的检测方法界定*MET*扩增的阈值均没有明确标准^[7]^。

#### 2.2.1 FISH

FISH法主要通过两种方式来定义*MET*扩增。其一依赖于测定*MET* GCN，现有临床研究多采用Cappuzzo标准（*MET* GCN≥5）来界定*MET*扩增^[8, 9]^，也有其他学者建议*MET* GCN≥6个^[10]^甚至是15个^[11]^才能定义为*MET*扩增，此法不足之处是无法区分多倍体和局部扩增。其二通过计算MET位点数目与7号染色体探针（chromosome 7 probe, CEP7）的比值作为评估*MET*扩增的依据（MET/CEP7≥2.0），这也是目前最广为接受的标准^[10]^。

#### 2.2.2 NGS

目前基于NGS的检测方法主要有两种：扩增法和杂交捕获法，两种方法各有利弊，相较于扩增法，杂交捕获法覆盖的基因组区域更加广泛，可识别和剔除重复序列，能够更准确地评估*MET*和其他基因的拷贝数变异^[12]^。然而使用NGS检测扩增时不仅需要考虑一些技术层面的问题，且NGS有时会出现难以解释的结果，目前尚无研究证实FISH法和NGS法的一致性，因此FISH法迄今为止被认为是相对更可靠的技术^[7]^。

#### 2.2.3 其他

IHC作为检测*MET*扩增的另一种方法，有临床研究对阳性的定义是评分为200或更高^[12]^，此外qRT-PCR也是一种检测*MET*扩增的方法，但是与FISH法、NGS方法相比，IHC、PCR法优势并不显著。

## 3 *MET*扩增——获得性耐药机制

### 3.1 EGFR抑制剂耐药与*MET*扩增

*MET*扩增是目前已经明确的EGFR-TKIs治疗获得性耐药的重要机制之一，属于旁路激活途径。5%-20%接受第一/二代TKIs治疗的患者会发生*MET*扩增介导的耐药^[13, 14]^。接受第三代TKIs奥希替尼治疗的患者，无论是一线还是后线治疗，发生*MET*扩增的比例大致相同，占10%-24%^[15]^。对于EGFR-TKIs耐药后*MET*扩增的患者，传统化疗^[16]^、MET抑制剂单药疗法^[17]^、免疫抑制剂疗法^[18]^等效果均有限。而EGFR-TKIs联合MET-TKIs的双通路抑制已经在多项临床研究^[16, 19, 20]^中显示出不错的疗效获益。

2021年美国临床肿瘤学会（American Society of Clinical Oncology, ASCO）年会上公布了由我国研究者开展的一项真实世界研究^[19]^，70例接受过EGFR-TKIs治疗进展后出现*MET*扩增的晚期NSCLC患者，接受了三种治疗方案：EGFR-TKIs联合克唑替尼（DT组，*n*=38）、克唑替尼单药治疗（ST组，*n*=10）、含铂双药化疗（CH组，*n*=22）。结果显示：DT组的客观缓解率（objective response rate, ORR）和疾病控制率（disease control rate, DCR）显著优于CH组（ORR: 48.6% *vs* 18.2%, *P*=0.026; DCR: 82.9% *vs* 50.0%, *P*=0.016），而与ST组相比，却无显著统计学差异（ORR: 48.6% *vs* 40.0%, *P*=0.73; DCR: 82.9% *vs* 70.0%, *P*=0.39）。生存分析结果显示：DT组的无进展生存期（progression-free survival, PFS）较ST组（5.0个月*vs* 2.3个月，*P*=0.004）及CH组（5.0个月*vs* 2.9个月，*P*=0.036）显著延长，然而，三组的总生存期（overall survival, OS）无显著差异（10.0个月*vs* 4.1个月*vs* 8.5个月，*P*=0.088）。此外，该研究还发现合并*TP53*突变及*EGFR*扩增的分子特征均不会影响EGFR-TKIs联合克唑替尼的疗效。

INSIGHT研究^[16]^是第一项比较替泊替尼联合吉非替尼和标准化疗用于MET过表达和*MET*扩增晚期NSCLC的多中心随机临床研究。研究结果显示，在接受替泊替尼和吉非替尼联合治疗的*MET*扩增患者中，近67%的患者在该研究的IB期和II期均取得了客观缓解，同时联合用药也是安全和可耐受的，联合治疗组及化疗组的中位PFS分别为16.6个月、4.2个月（HR=0.13），中位OS分别为37.3个月、13.1个月（HR=0.08），该研究还强调了替泊替尼联合吉非替尼对*MET*高水平扩增患者的疗效更加显著。

2021年世界肺癌大会（World Congress of Lung Cancer, WCLC）上，发布了国际多中心TATTON研究^[20]^的部分结果，该研究入组既往接受EGFR-TKIs治疗后出现*MET*扩增的局部晚期或转移性NSCLC患者。扩展队列的结果表明奥希替尼联合赛沃替尼安全性良好，同时对于既往未经过三代EGFR-TKI治疗耐药后出现*MET*驱动突变的NSCLC患者疗效确切，其中对于伴有T790M阳性的患者，疗效最为显著（PFS：11.1个月，ORR=67%）。此外，该研究中B1部分显示，既往经过三代EGFR-TKI治疗的患者使用联合方案疗效并不显著（PFS：5.5个月，ORR=33%），可能与既往接受多线治疗及较重的疾病转移负担有关。

2021年欧洲肿瘤内科学会年会（European Society for Medical Oncology, ESMO）公布了II期ORCHARD研究^[21]^A组部分（*MET*基因变异组）疗效数据，入组的20例患者均接受奥希替尼联合赛沃替尼的治疗方案，17例可评估疗效，其中7例患者经研究者评估达到了客观缓解，其ORR达到了41%（95%CI: 25%-59%），体现了初步的抗肿瘤活性，同样并未发现新的安全性事件。

*Science Translational Medicine*上发表了一项临床前研究^[22]^，与我们传统认知相悖，表明部分同时存在*EGFR*突变和*MET*扩增的肺癌患者，可能仅依赖于MET的激活，对于这部分人群，仅需要使用单药MET-TKIs治疗，而并非目前所认可的EGFR-TKIs联合MET-TKIs的治疗方案。研究^[22]^进一步发现对MET-TKIs单药治疗敏感的细胞相较于对EGFR-TKIs敏感的细胞以及EGFR-TKIs和MET-TKIs联合处理敏感的细胞，其EGFR: MET mRNA的比率更低，临床中似乎可以通过评估EGFR: MET mRNA的比率来预测其对MET-TKIs单药治疗的敏感性。因其仅是一项临床前研究，结论尚需在未来更多的前瞻性临床试验中来验证。

### 3.2 间变性淋巴瘤激酶（anaplastic lymphoma kinase, ALK）抑制剂耐药与*MET*扩增

*ALK*融合是NSCLC中又一种常见的分子亚型，目前，ALK抑制剂已经发展到第三代，与EGFR-TKIs类似，克唑替尼等ALK抑制剂虽疗效显著，但同样面临着耐药的问题，研究^[23, 24]^发现约50%的NSCLC患者对ALK-TKIs的耐药是通过非ALK依赖途径实现的，其中最常见的原因是旁路信号途径的激活，包括MET、EGFR、SRC和IGF-1R等。

Shi等^[25]^建立了对ALK抑制剂耐药的晚期肺腺癌来源的人源性肿瘤组织异种移植（patient-derived xenograft, PDX）模型，全外显子测序证实，在PDX模型中，*MET*扩增是阿来替尼的独立耐药机制，ALK抑制剂联合MET抑制剂的双通路抑制在PDX模型克服耐药中十分有效。Ji等^[26]^报道了1例*ALK*阳性肺腺癌患者，一线接受阿来替尼治疗6个月后疾病进展，肾上腺转移灶活检组织NGS检测提示：*MET*高水平扩增，未发现*ALK*突变，给予克唑替尼治疗10个月后再次进展，二次肾上腺活检行MET FISH检测示：MET/CEP7=9.6，同时循环肿瘤DNA（circulating tumor DNA, ctDNA）NGS检测提示：*EML4-ALK*阳性，且未发现其他耐药突变。随后给予阿来替尼联合克唑替尼的联合治疗策略，2周后随访行正电子发射计算机断层显像（positron emission tomography-computed tomography, PET-CT）检查示：肾上腺、纵隔及胸膜的转移性病灶较前显著改善，直到4个月后患者出现颅内转移性病灶。Dagogo-Jack等^[27]^分析了来自136例*ALK*阳性NSCLC患者ALK-TKIs治疗后的207个组织（*n*=101）和血浆（*n*=106）样本，采取FISH检测或NGS检测来评估*MET*扩增的基因改变。结果显示，11例（13%）患者的活检组织样本检测出*MET*扩增，相较于克唑替尼耐药后二线接受第二/三代ALK抑制剂的患者，一线接受第二/三代ALK-TKIs治疗的患者更容易出现*MET*扩增的基因改变（33% *vs* 9%, *P*=0.019）。该研究^[27]^进一步在临床前模型中证实了MET-TKIs卡马替尼或赛沃替尼可部分抑制细胞增殖，而劳拉替尼和卡马替尼或赛沃替尼的联合使用抑制细胞增殖的效力更强，表明ALK/MET的双重阻断可强效抑制ALK及其下游的信号通路。基于上述结果，本研究中2例因MET改变而发生获得性耐药的患者，接受劳拉替尼联合克唑替尼的治疗方案后迅速获益，进一步验证了双靶联合方案在ALK抑制剂耐药后出现MET改变患者中的治疗潜力，但最佳联合方案仍需在更多的前瞻性临床试验中进行评估。

### 3.3 *c-ros*原癌基因1（c-ros oncogene 1, ROS1）抑制剂耐药与*MET*扩增

*ROS1*重排占NSCLC的1%-2%，已逐渐成为NSCLC的重要致癌驱动基因以及治疗靶点之一^[28, 29]^。随着研究的深入，越来越多的ROS1抑制剂已被获批用于临床。目前关于*ROS1*重排NSCLC对TKIs耐药机制的研究中，已发现了*ROS1*激酶结构域外显子的继发突变。而随着恩曲替尼和劳拉替尼在美国和日本的上市，其耐药模式又有了新的发现。

Lin等^[30]^对晚期*ROS1*融合阳性NSCLC患者接受克唑替尼或劳拉替尼治疗进展后的组织或血浆样本进行了重复基因检测，以探索其耐药机制，结果表示在部分未发现*ROS1*激酶结构域耐药突变的患者中，可能存在ROS1非依赖性耐药机制，2例患者的组织NGS检测结果提示出现了*MET*扩增，1例为治疗前胸水FISH检测提示未见*MET*扩增（FISH: MET/CEP7=1），接受劳拉替尼治疗耐药后的脑转移灶组织NGS检测结果提示：*MET*扩增（FISH: MET/CEP7=6.3），另一例患者的肾上腺转移灶组织NGS检测同样出现了*MET*扩增（FISH: MET/CEP7 > 25），提示*MET*扩增可能为ROS1非依赖性耐药机制。Yang等^[31]^报道了1例*ROS1*重排转移性肺腺癌患者，一线接受克唑替尼治疗19个月后，脑转移灶迅速进展（期间给予劳拉替尼治疗，脑转移相关症状较前加重），随后进行了紧急的脑转移灶手术，术后病理标本NGS检测回报提示：高水平*MET*扩增（GCN: 32），为进一步验证*MET*扩增为TKIs治疗后出现，研究者们又对肺部原发灶再次进行了FISH检测，结果并未检测出*MET*扩增。该研究同样提示*MET*扩增可能为ROS1-TKIs的潜在耐药机制，而遗憾的是，该患者最终死于严重脑水肿和脑疝，未尝试ROS1-TKIs联合MET-TKIs的联合治疗策略。目前尚无公开的研究数据表明ROS1抑制剂耐药后*MET*扩增的患者中双靶联合治疗策略的可行性与安全性，未来有必要继续探索ROS1非依赖的耐药机制及应对策略。

### 3.4 选择性转染重排（rearranged during transfection, RET）抑制剂耐药与*MET*扩增

我国NSCLC患者中*RET*基因融合的发生率占1.4%-2.5%^[32]^。塞尔帕替尼（Selpercatinib）和普拉替尼（Pralsetinib）是特异性RET抑制剂，已被美国食品药物监督管理局（Food and Drug Administration, FDA）批准用于转移性*RET*融合阳性的NSCLC成人患者。根据NSCLC靶向治疗的经验，耐药的发生是不可避免的，目前RET抑制剂耐药相关的分子机制尚不完全清楚。

Lin等^[33]^对一组*RET*融合阳性NSCLC患者接受塞尔帕替尼和普拉替尼治疗耐药后的肿瘤组织和血浆进行了详细分析，其中3例（15%）耐药患者检测出*MET*扩增，2例（10%）检测出*RET*耐药突变（为已知的RET G810S和G810C突变），1例检测出*KRAS*扩增。由此可见，*MET*扩增可能是这些新型RET抑制剂的潜在耐药机制。Rosen等^[34]^的一项研究进一步在体外模型中表明，*MET*扩增是塞尔帕替尼获得性耐药的潜在机制，且塞尔帕替尼和克唑替尼的联合治疗可以克服其耐药性。同样该研究中4例接受塞尔帕替尼耐药的NSCLC患者，二次组织NGS检测提示*MET*扩增，鉴于联合使用EGFR和MET抑制剂可以有效克服MET驱动的耐药性已被大家所熟知，研究者们随后采取了尝试塞尔帕替尼联合克唑替尼的双靶抑制策略，结果也证实了这种联合治疗的有效性，其中1例患者的治疗缓解时间更是达到了10个月。遗憾的是，该研究的病例数（*n*=4）太少。然而Meng等^[35]^报道了1例*RET*融合阳性（*KIF5B-RET*, *FOXD1-RET*）IV期肺腺癌患者，一线接受贝伐珠单抗联合培美曲塞和卡铂治疗14个月后进展，二线接受塞尔帕替尼靶向治疗9个月后再次进展，二次基因检测提示*MET*扩增，随后接受了塞尔帕替尼联合克唑替尼的双靶治疗方案，然而患者的一般状况迅速恶化，4个月后死于癌症。因此，这更加凸显了未来进行更大规模前瞻性研究的重要性，以探索RET抑制剂联合MET抑制剂的疗效及安全性，为我们带来更加准确的循证医学证据。

### 3.5 Kirsten大鼠肉瘤（Kirsten-RAS, KRAS）抑制剂耐药与*MET*扩增

*KRAS*是一种癌症常见突变基因，2021年5月，FDA批准Sotorasib（AMG 510）^[36]^用于治疗先前已经接受过至少一种系统疗法、经FDA批准的检测方法证实存在*KRAS*^G12C^突变、局部晚期或转移性NSCLC成人患者。其临床研究CodeBreak（NCT03600883）^[37]^在后线治疗中ORR达到了36%，中位PFS为6.8个月，中位OS为12.5个月。同样，探索耐药突变谱至关重要，但CodeBreak研究^[37]^中尚未报道连续活检数据和连续ctDNA数据。

在Awad等^[38]^的研究中，10例被评估为疾病进展的*KRAS*^G12C^突变NSCLC患者中，2例（20%）患者中发现了*MET*扩增。同样另一项研究^[39]^发现，在23例对*KRAS*^G12C^抑制剂Adagrasib产生耐药的NSCLC患者中，2例患者检测出*MET*扩增，并未发现其他可能的耐药机制，包括*KRAS*继发性突变等。Suzuki等^[40]^在构建的耐Sotorasib的*KRAS*^G12C^NSCLC细胞株中检测到了*MET*扩增的亚克隆进化，深入探索发现*MET*扩增既可以促使RAS由非活性状态向活性状态转换，通过激活RAS-RAF-MEK-ERK途径来实现对Sotorasib的耐药，还可以通过RAS非依赖性途径激活AKT来诱导对*KRAS*^G12C^抑制剂的耐药性。且在细胞株模型中，MET抑制剂克唑替尼可通过抑制Ras-MEK-ERK和AKT信号通路来恢复对Sotorasib的敏感性；在耐Sotorasib的异种移植肿瘤小鼠模型中，MET抑制剂及*KRAS*^G12C^抑制剂的联合治疗可使移植瘤缩小，且并未发现毒性作用。因此，*MET*扩增作为选择性小分子*KRAS*^G12C^抑制剂耐药可能的驱动因素，双通路阻断同样有希望成为克服*MET*扩增所介导*KRAS*^G12C^突变NSCLC耐药性的一种有前景的治疗选择。

### 3.6 神经营养因子受体酪氨酸激酶（neurotrophic tyrosine receptor kinase, NTRK）抑制剂与*MET*扩增

*NTRK*基因融合作为NSCLC的一个独特分子亚型，与获批药物数量众多的EGFR等靶点相比，患者生存情况并不乐观。原肌球蛋白受体激酶（tropomyosin receptor kinase, TRK）抑制剂作为针对*NTRK*基因融合的靶向治疗药物，为无法使用常规靶向治疗药物的患者提供了新的选择^[41]^。有研究^[42]^表明，对TRK抑制剂的耐药性是由丝裂原活化蛋白激酶（mitogen-activated protein kinase, MAPK）通路的基因组改变所介导的，而*MET*扩增已被确定为耐药的关键。目前，关于*NTRK*融合阳性肺癌患者TKIs治疗后耐药的研究报道相对较少，Cocco等^[42]^报道了1例*NTRK*融合阳性的胆管癌患者，一代TRK抑制剂耐药后检测出*MET*扩增，且对NTRK的测序并未发现激酶结构域点突变。

## 4 小结与展望

*MET*扩增是目前已经明确的*EGFR*阳性NSCLC获得性耐药的机制之一，而近年来，越来越多的研究发现，MET扩增不仅存在于*EGFR*阳性患者中，而且在*ALK*、*RET*、*ROS1*等驱动基因阳性NSCLC患者的获得性耐药机制中同样发挥着关键作用。目前，在*EGFR*阳性NSCLC患者中，EGFR和MET的双通路抑制在国内外几项大型临床研究中均取得了不错的疗效和安全性数据；在*ALK*、*RET*、*ROS1*等驱动基因阳性NSCLC患者中，通过前文所述不难发现，双靶联合治疗策略也有望成为克服*MET*扩增所介导获得性耐药的最佳治疗选择之一。因此，未来期待有更多的前瞻性临床试验来继续验证该双靶联合治疗策略的疗效和安全性。精准治疗时代下，随着MET抑制剂的不断推陈出新，相信在不久的将来，不同的药物组合可以最大化发挥其疗效。
